# Effects of Different Proportions of Cattle Manure and Mushroom Residue on Yield and Quality of Cucumber Fruit

**DOI:** 10.3390/plants14091371

**Published:** 2025-04-30

**Authors:** Ruochen Wang, Ziyi Wang, Zhaomei Liu, Tingting Zhang, Shuxia Chen

**Affiliations:** 1 College of Horticulture, Northwest A&F University, Xianyang 712100, China; 2 Shaanxi Engineering Research Center for Vegetables, Northwest A&F University, Xianyang 712100, China

**Keywords:** cattle manure, mushroom residue, yield, quality, cucumber fruit

## Abstract

Large-scale agricultural and animal husbandry production in Shaanxi Province of China has led to significant environmental pollution, due to the incineration of vast amounts of agricultural waste annually. As the land area used for vegetable cultivation expands and farming practices evolve, the demand for organic substrates continues to grow. To optimize cost savings and enhance efficiency, this study investigated the effects of different organic substrate compositions on cucumber (*Cucumis sativus*) yield and quality, using ‘Jinyou 35’ cucumber as the experimental model. The results demonstrated that the blended organic substrates derived from agricultural waste met key physicochemical requirements for cucumber cultivation across both seedling establishment and fruit development stages. Compared with the control, the T4 treatment (mushroom residue/cattle manure = 1:1) increased the cucumber yield and its content of total sugar, vitamin C, and fatty acids. Furthermore, the T6 treatment (mushroom residue/cattle manure = 3:1) produced the highest total aroma and the lowest soluble protein content compared to the other treatments, and the level of C6 aldehydes in the cucumber fruits was significantly higher (*p* < 0.05) in this treatment group than in the control group. The findings suggest that properly formulated organic substrate blends can serve as effective growing media for cucumber cultivation, while simultaneously mitigating environmental pollution. This study provides a theoretical foundation for the sustainable utilization of agricultural waste-derived organic substrates in vegetable production.

## 1. Introduction

The rapid expansion of facility horticulture has driven a surge in demand for high-quality cultivation substrates to support vegetable production in protected agricultural systems. Commercial substrates are currently dominated by non-sustainable components, predominantly peat supplemented with vermiculite and perlite [1,2]. Although peat demonstrates favorable organic properties, its high economic costs and non-renewable nature critically hinder its application for the sustainable development of modern horticultural operations [3].

Agricultural waste is diverse, and many of its by-products can serve as alternative cultivation substrates [4,5]. In developed countries such as Canada, the UK, and Israel, agricultural residues such as sawdust, cattle manure, grape pomace, and coconut coir have been successfully utilized as substitutes for peat-based substrates, demonstrating excellent performance in vegetable cultivation [6,7]. For example, coconut coir has been used as a peat alternative in oak tree (*Quercus*) cultivation [8], while sediment accumulated at the bottom of rivers, after undergoing disinfection and decontamination, has been employed as a cultivation substrate [9]. Studies indicate that using agricultural waste as a peat substitute in plant production can effectively reduce the incidence of plant diseases [10]. Various cotton (*Gossypium hirsutum*) by-products have yielded results comparable to those of peat-based substrates [11], and rice (*Oryza sativa*) husks, a common agricultural waste, have been successfully used as a substrate for ornamental plants such as roses, partially replacing peat in horticultural applications [12]. The development of agricultural waste-based substrates could substantially reduce the demand for peat in vegetable cultivation, while lowering production costs, improving industry profitability, and addressing issues related to resource wastage and environmental pollution. Meanwhile, the development of a new cultivation substrate is also aligned with the requirements of soilless culture outlined in the FAO Plant Production and Protection Paper (2013) and the EU 2030 environment and climate plans. As a medium for material exchange between plant roots and the external environment, the properties of a cultivation substrate directly influence plant water and nutrient absorption, affecting seedling growth, fruit yield, and overall crop quality [13,14,15].

Cucumber (*Cucumis sativus*) is one of the most widely cultivated vegetables globally [16,17]. Fruit development is influenced by a series of internal factors, such as plant hormones, and external environmental factors, such as temperature, light, and soil. These factors regulate fruit development by modulating the expression of various growth- and development-related factors and genes [18,19,20]. However, long-term continuous cropping in facility-based cucumber production has led to frequent occurrences of pests and diseases, reduced yield and quality, soil salinization, and degradation [21,22,23,24]. The use of agricultural waste as a cultivation substrate is a promising strategy for reducing fertilizer dependency, alleviating soil resource constraints, improving crop yield and quality, and lowering substrate costs [25,26]. Despite their potential, research on the optimization of agricultural waste-based substrates remains limited, necessitating further systematic investigation.

In this study, locally abundant agricultural waste materials, including cattle manure and mushroom residue, were mixed in specific ratios to formulate a cultivation substrate for cucumber production. By comparing the effects of these substrate formulations with those of commercially available substrates, we aimed to evaluate their impact on cucumber fruit yield and quality. The findings of this study provide a scientific basis for the practical application of agricultural waste-based substrates in facility vegetable production, contributing to the development of sustainable and cost-effective cultivation practices.

## 2. Results

### 2.1. Physical and Chemical Properties of Different Substrates of Cattle Manure and Mushroom Residue

In this study, the physical and chemical properties of composite substrates composed of different ratios of cattle manure and mushroom residue were analyzed (Table 1). The composite substrates exhibited a bulk density ranging from 0.39 to 0.46 g/cm^3^, a total porosity between 52.03% and 55.48%, a water-holding porosity ranging from 45.37% to 47.85%, pH values ranging from 6.90 to 7.22, and an electrical conductivity (EC) ranging from 3.33 to 7.13 mS/cm. Among the treatments, T1 had the highest bulk density, while T7 had the lowest, with all treatments showing significantly higher values than the control (*p* < 0.05). The water-holding porosity was highest in T7 and lowest in T1. Additionally, T1 had the lowest pH and the highest EC, both significantly greater than the control (*p* < 0.05). The results indicate that as the proportion of cattle manure decreased and the proportion of mushroom residue increased, the total porosity, aeration porosity, gas–water ratio, and pH increased, whereas the EC decreased.

### 2.2. Effect of Mixed Substrates of Cattle Manure and Mushroom Residue on Cucumber Yield

In the spring crop of 2020, the T3 treatment (mushroom residue/cattle manure = 3:5) achieved the highest yield per plant and per acre (Table 2), exceeding that of the control by 69.69%. The lowest yield was observed in the T1 treatment, which was still 0.93 times higher than that of the control. For the spring crop of 2021, the T6 treatment recorded the highest single-plant yield and total yield per mu, at 3.06 kg and 4281.86 kg, respectively, representing an increase of 21.05% over the control. The lowest yield among the different substrate compositions was observed in the T3 treatment (3535.57 kg), which was comparable to that of the control. This suggests that appropriate ratios of cattle manure and mushroom residue can effectively serve as cultivation substrates to enhance cucumber yield. Furthermore, the yields in the T4 to T6 treatments were higher than those of the control, indicating that a cattle manure-to-mushroom residue ratio within the range of 1:1 to 1:3 is optimal for use as a cultivation substrate.

### 2.3. Influence of Mixed Substrates on Nutritional Quality of Cucumber Fruit

**Figure 1:**
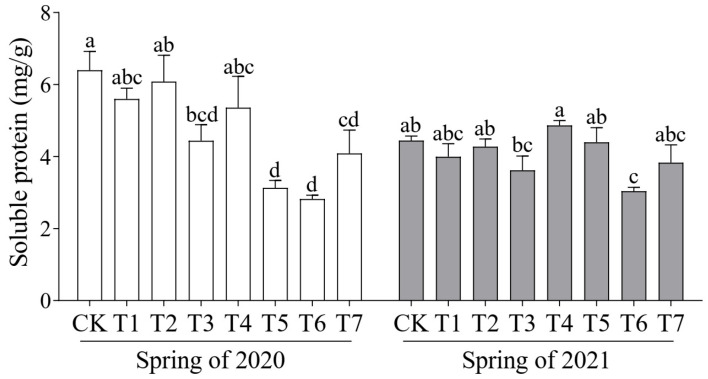
Effects of different proportions of cattle manure and mushroom residue on soluble protein in cucumber fruit. Different lowercase letters represent significant differences at 0.05 level.

**Figure 2:**
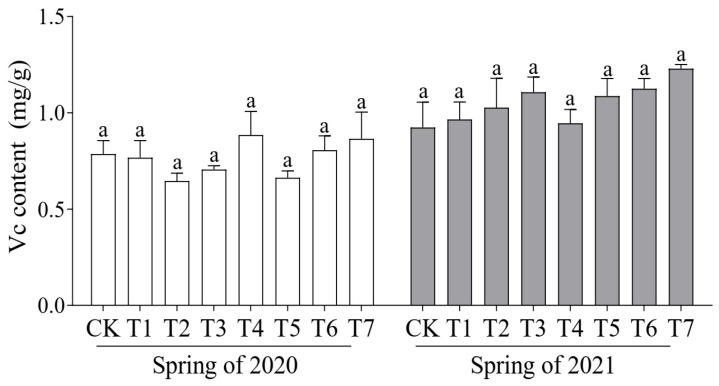
Effects of different proportions of cattle manure and mushroom residue on Vc content of cucumber fruit. Different lowercase letters represent significant differences at 0.05 level.

Soluble protein and vitamin C (Vc) are key indicators of the nutritional quality of cucumber fruit; therefore, their content was measured across the different treatments. In the spring crop of 2020, the soluble protein content of all treatments was lower than that of the control, with the highest level observed in the T2 treatment (6.08 mg/g). The highest Vc content (0.88 mg/g) was recorded in the T4 treatment (Figure 1). In the spring crop of 2021, T4 exhibited the highest soluble protein content (5.15 mg/g), while the Vc content was higher than that of the control across all treatment groups, with T7 exhibiting the highest Vc content of 1.23 mg/g (Figure 2).

**Figure 3:**
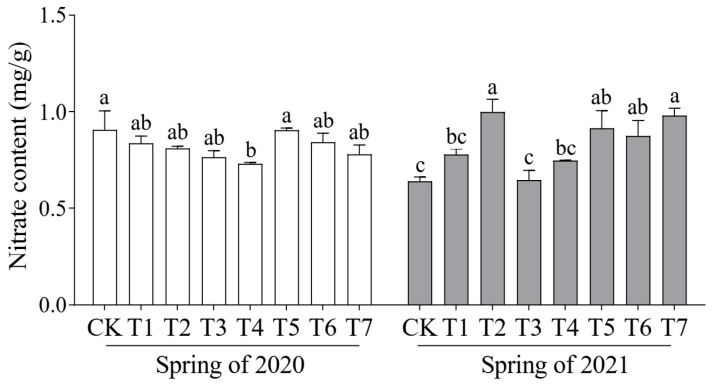
Effects of different proportions of cattle manure and mushroom residue on content of nitrate in cucumber fruit. Different lowercase letters represent significant differences at 0.05 level.

Although nitrate is not an essential nutrient for humans, its excessive intake may pose health risks. Therefore, monitoring nitrate content is critical as a quality control parameter in vegetable production. In the spring crop of 2020, T4 had the lowest nitrate content among all treatments (0.73 mg/g) (Figure 3). In the spring crop of 2021, the nitrate content across treatments ranged from 1.05% to 56.34% higher than the control, with the lowest level recorded in the T3 treatment (0.64 mg/g), which was different from those of the other treatments and the control. Overall, the T4 treatment increased the Vc content by 12.52% in 2020 and 2.4% in 2021 compared to the control, while maintaining a relatively low nitrate content. These findings suggest that a moderate increase in mushroom residue can enhance Vc accumulation and improve the overall nutritional quality of cucumber fruit.

### 2.4. Effect of Mixed Substrates of Cattle Manure and Mushroom Residue on Soluble Sugars of Cucumber Fruit

The primary soluble sugars in cucumber fruit include fructose, glucose, and sucrose. In this study, their content was analyzed at the harvest stage (Table 3). In the spring crop of 2020, the T4 treatment exhibited the highest fructose and glucose content, with increases of 4.27% and 0.47% compared to the control, respectively. Meanwhile, the T2 treatment recorded the highest sucrose content, showing a 37.11% increase compared to the control. In the spring crop of 2021, the T7 treatment had the highest fructose and glucose content, increasing by 9.89% and 41.15% compared to the control, respectively. The highest sucrose content was observed in the T2 treatment, with a 63.58% increase compared to the control. These results indicate that the fructose and sucrose accumulation in the cucumber fruits increased as the proportion of cattle manure decreased and that of mushroom residue increased.

### 2.5. Effects of Different Proportions of Cattle Manure and Mushroom Residue on Fatty Acids of Cucumber Fruit

The primary saturated fatty acids in cucumber fruit are palmitic and stearic acids, while the main unsaturated fatty acids are linoleic and linolenic acids (Table 4). In the spring crop of 2020, the T4 treatment exhibited the highest palmitic acid (76.10 μg/g) and linoleic acid (180.07 μg/g) content. The highest stearic acid content (51.17 μg/g) was recorded in the T3 treatment, while the highest linolenic acid content was observed in the T6 treatment. In the spring crop of 2021, the highest saturated fatty acid content was found in the T6 treatment, with an increase of 29.43% for palmitic acid content and 53.42% for stearic acid content compared to those of the control. The highest linoleic (600.92 μg/g) and linolenic (259.48 μg/g) acid content was recorded in the T3 treatment. Data from both years indicate that fatty acid accumulation in cucumber fruit was maximized in the T3 to T6 treatments, suggesting that reducing cattle manure and increasing mushroom residue in the substrate composition can enhance fatty acid content.

### 2.6. Analysis of Volatile Compounds of Cucumber Fruit in Different Treatments

The volatile compounds in cucumber fruit primarily include ketones, aldehydes, alcohols, and esters. In this study, the aromatic content of cucumber fruit was analyzed using solid-phase microextraction (SPME) combined with gas chromatography–mass spectrometry (GC-MS).

**Figure 4:**
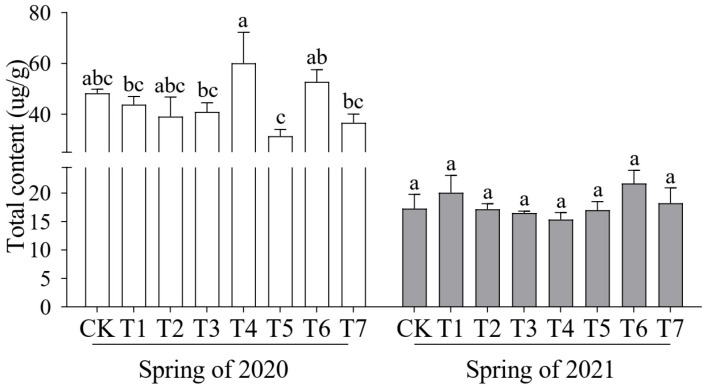
Effects of different proportions of cattle manure and mushroom residue on total aroma content of cucumber fruit. Different lowercase letters represent significant differences at 0.05 level.

The different composite substrates led to significant variations in total aroma content. In the spring crop of 2020, the T4 treatment exhibited the highest total aroma content (60.05 μg/g). However, in the spring crop of 2021, no significant differences were observed among treatments, with the highest aroma content recorded in the T6 treatment (21.69 μg/g) (Figure 4).

**Figure 5:**
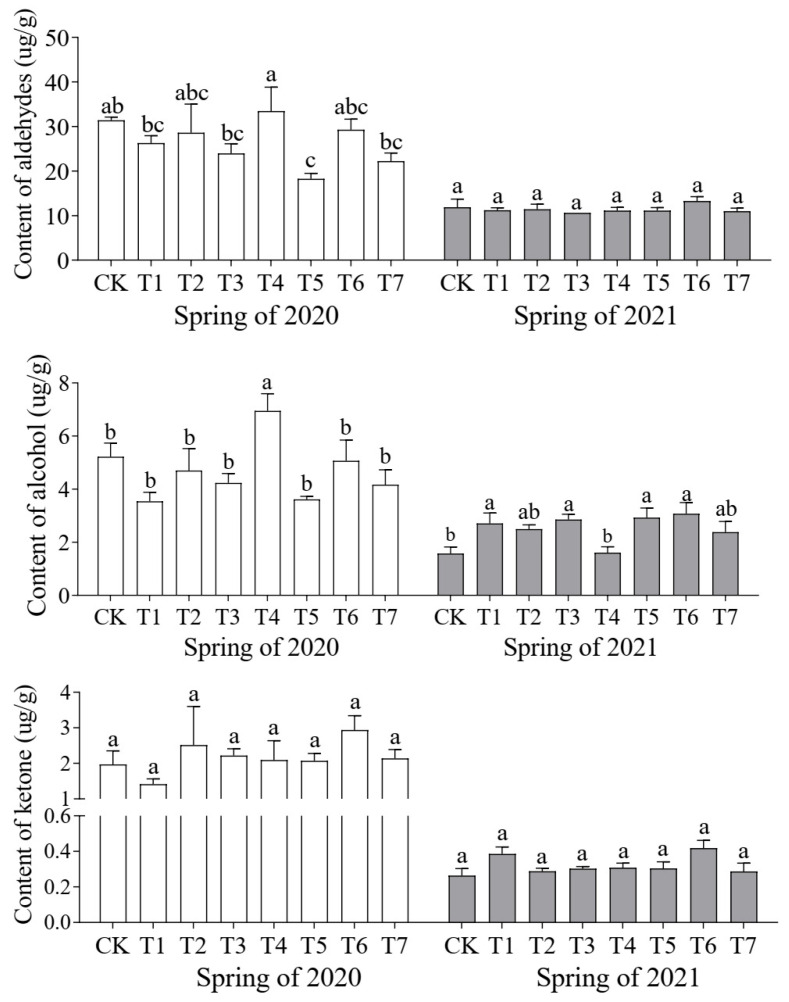
Effects of different proportions of cattle manure and mushroom residue on content and species of volatiles in cucumber fruit. Different lowercase letters represent significant differences at 0.05 level.

The composition of aromatic compounds in cucumber fruit across treatments was largely similar, with aldehydes being the predominant volatiles, accounting for 55.5% to 65.02% of the total aroma content (Figure 5). Significant differences were observed in the content of individual aroma compounds. In 2020, the highest aldehyde content was found in the T4 treatment (33.45 μg/g), which was higher than that of the control, followed by the T6 treatment (29.28 μg/g). In 2021, the highest aldehyde content was recorded in the T6 treatment.

**Figure 6:**
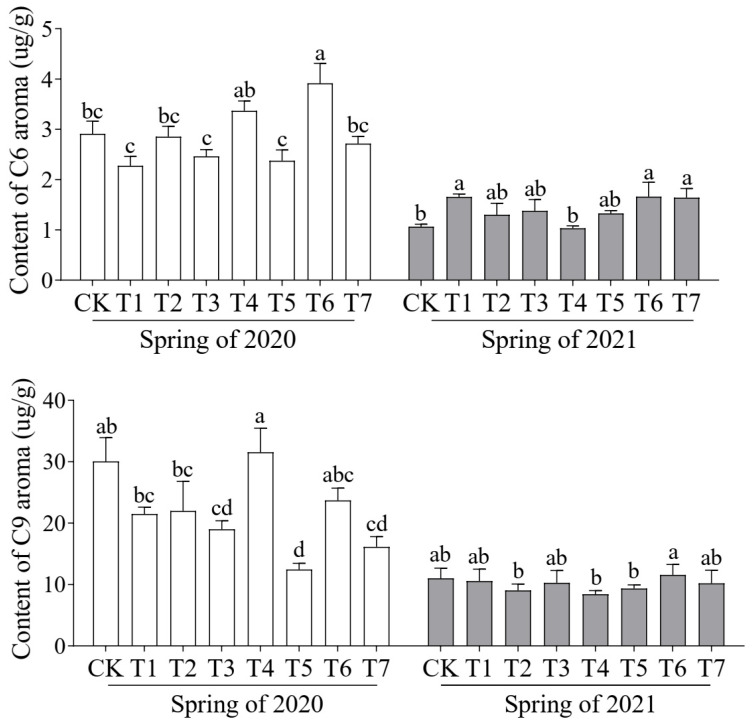
Effects of different proportions of cattle manure and mushroom residue on content of C6 and C9 volatiles in cucumber fruit. Different lowercase letters represent significant differences at 0.05 level.

C6 and C9 aldehydes are the key contributors to the characteristic aldehydic aroma of cucumber, and play a crucial role in its flavor profile. In the spring crop of 2020, the highest C6 aldehyde content (3.92 μg/g) was observed in the T6 treatment, while the highest C9 aldehyde content (31.54 μg/g) was found in the T4 treatment (Figure 6). In 2021, both C6 and C9 aldehydes reached their highest levels in the T6 treatment.

These findings suggest that different ratios of cattle manure and mushroom residue in the substrate composition significantly influence the total aroma content and the accumulation of C6 and C9 aldehydes in cucumber fruit. Among all treatments, the T6 treatment was the most effective in promoting the biosynthesis of C6 aldehydes.

## 3. Materials and Methods

### 3.1. Plant Materials and Treatments

The experiment used cattle manure and mushroom residue, mixed with vermiculite and perlite to create 7 treatments, where both vermiculite and perlite accounted for 10% each of the total volume, while the mixture of mushroom residue and cattle manure comprised 80% of the total volume. The volume ratios of mushroom residue to cattle manure for treatments T1 to T7 were as follows: 1:7, 1:3, 3:5, 1:1, 5:3, 3:1, and 7:1; a commercial substrate was used as a control (CK). The CK substrate was purchased from Xinxian Luyuan Seedling Substrate Company in Shandong Province, China. The composting process of the mixed substrates was conducted through aerobic fermentation over a 30-day period. Mechanical turning was performed at 5-day intervals to ensure oxygen homogeneity and uniform decomposition. Temperature profiles were systematically monitored using embedded probes, with a thermophilic phase (55–65 °C) deliberately maintained for 7 consecutive days to ensure pathogen inactivation and weed seed eradication. The moisture content was regulated within the 60–65% range throughout the periodic water supplementation. All treatments underwent identical composting protocols prior to subsequent application in cultivation trials of different years.

The cucumber variety used in this study was the local main cultivar ‘Jin You 35’. Healthy cucumber seeds were selected and soaked in warm water (55 °C) for 20 min, and then immersed in room-temperature water for 4 h on 3 March 2020 and 6 March 2021. After the seeds had absorbed sufficient water, they were placed in petri dishes lined with filter paper, and germinated at a constant temperature of 25 °C. Once two-thirds of the seeds had begun to sprout, they were sown into a seedling tray for further cultivation on 5 March 2020 and 8 March 2021. One seed was sown per cell, and three trays were used for each treatment. After sowing, a uniform layer of vermiculite was applied to cover the seeds, followed by thorough watering. The cucumber was cultivated under a 16/8 h light/dark cycle, at 25 °C, in a light-controlled growth chamber. The relative water-holding capacity of the substrate was maintained between 85% and 95%, and the relative humidity of the air was kept between 80% and 90%.

Seedlings were transplanted at the 3-leaf stage on 31 March 2020 and 6 April 2021, and the greenhouse was controlled at about 25 °C after transplanting, with a planting density of 5 plants/m^2^. A completely randomized design with 8 substrate treatments × 3 replicates (24 units, 50 plants/unit) was used. Crop management included automated drip irrigation, weekly foliar water-soluble fertilizer supplementation, and biological pest control. At the full flowering stage on 30 April 2020 and 4 May 2021, female flowers at the 14th node were tagged at anthesis (designated as 0 days post-anthesis (DPA)). Undamaged, pest/disease-free labeled fruits were collected at 9 DPA, immediately ice-transported, flash-frozen in liquid nitrogen, and stored at −80 °C.

All procedures involving organic waste and plant cultivation complied with institutional and local environmental guidelines.

### 3.2. Physicochemical Properties of Substrates

The analyzed physicochemical properties of the substrates included bulk density, total porosity, aeration porosity, water-holding porosity, gas–water ratio, pH, and electrical conductivity (EC). After thoroughly mixing the composite substrate, samples were taken using the quartering method, with three replicates per treatment. Bulk density and porosity were calculated according to previously described methods [27]. Water-holding porosity was defined as the difference between total porosity and aeration porosity, while the gas–water ratio was the ratio of aeration porosity to water-holding porosity [28]. pH and EC were measured following the method described by Kratz et al. [29].

### 3.3. Determination of Cucumber Yield

For each replicate, 15–20 uniform cucumber plants were selected and labeled with tags. Yield measurements began when the cucumber fruit reached the commercial maturity stage, and the weight and harvest date were recorded. The individual plant yield was calculated, and the yield per acre was also determined.

### 3.4. Measurement of Cucumber Quality

Fruits from each treatment were collected to measure soluble protein, vitamin C, and nitrate content. The methods for determining nutritional quality followed previously described methods [30].

The sugar content of cucumber fruit was determined by following the sample preparation method [31]. A GC-MS system (Thermo Fisher, Austin, TX, USA) was used for automatic injection, and the chromatographic conditions and column specifications were based on a previously described method [32].

The determination of volatile compounds in cucumber fruit was carried out according to the method of Sun et al. [33]. An SPME-GC-MS system (Thermo Fisher, Austin, TX, USA) was used for manual injection. The chromatographic conditions and column specifications were based on the methods of Wang et al. [34].

The fatty acid content of cucumber fruit was determined by the sample preparation. An Agilent 7890B gas chromatograph (Agilent, Santa Clara, CA, USA) was used for measurement. The chromatographic conditions and column specifications were based on the method of Rudolph et al. [35].

### 3.5. Statistical Analysis

The data were processed and statistically analyzed using Microsoft Excel 2019 and SPSS 25. The experimental data represent the mean values of three replicates with standard deviations. Tukey’s multiple comparison test was used to detect differences between treatments at *p* < 0.05.

## 4. Discussion

### 4.1. Effects of Different Proportions of Cattle Manure and Mushroom Residue on Physical and Chemical Properties of Agricultural Waste-Based Substrates

The analyzed physicochemical properties of the substrates included bulk density, total porosity, pH, aeration porosity, water-holding porosity, gas–water ratio, and electrical conductivity (EC). These properties are closely related to the plant root system’s ability to absorb water and nutrients, thereby influencing plant growth, yield, and the quality of fruit. The bulk density of the substrate, defined as the mass of completely air-dried substrate per unit volume, reflects the compaction of the substrate. A high bulk density can cause compaction stress on the plant, making it difficult for the roots to grow downward, impairing the plant’s ability to absorb water and nutrients, and consequently affecting the growth of the above-ground part, which manifests as reduced plant height, stem diameter, and above-ground dry and fresh weights [36]. The electrical conductivity (EC) value of the substrate reflects its salinity, which is related to the nutrient supply for cucumber seedlings. Within an appropriate range, an increase in EC can promote cucumber growth [37]. High EC can hinder normal water and nutrient uptake, resulting in slow above-ground growth, leaf edge curling, and stiffening of leaves. Each plant species has an optimal pH range for growth, with the optimal pH for cucumber seedlings being between 5.7 and 7.2 [38]. As shown in Table 1, the commercial substrate and the agricultural waste-based substrates used in this study generally meet the pH requirements for cucumber seedling and fruit growth. When using agricultural waste to prepare cultivation substrates, it is important to consider both the advantages and disadvantages of the raw materials. Methods such as crushing, sieving, and leaching to reduce salt content should be employed to improve the physicochemical properties of the components, in order to achieve better plant growth, yield, and quality of fruit.

### 4.2. Effects of Different Proportions of Cattle Manure and Mushroom Residue on Cucumber Yield

Compared to soil-based cultivation, the use of agricultural waste substrates can enhance cucumber yield and quality [39]. Substrates mixed with mushroom residue and medicinal plant residues as primary organic materials have been shown to increase cucumber yield, and an optimal ratio of mushroom residue and crop straw can further improve fruit yield. The addition of zeolite and perlite to rice husk or sawdust substrates has been reported to significantly increase chili pepper yield [40]. Incorporating rice husk and biochar into the substrate has also demonstrated beneficial effects on cucumber growth and yield [41]. Additionally, the inclusion of sheep wool in the substrate resulted in a 33% increase in tomato and chili pepper yields, with similar benefits observed in cucumbers [42,43].

In this study, the T4 treatment (mushroom residue/cattle manure = 1:1) showed relatively high yields in the two spring crops, with increases of 37.66% and 16.70%, respectively, compared to the control. These results suggest that varying substrate ratios significantly influence cucumber yield, aligning with previous research findings. Moreover, when comparing the spring cucumber yields across two years, the yield variations across treatments indicate that environmental factors play a role in plant growth.

### 4.3. Effects of Different Proportions of Cattle Manure and Mushroom Residue on Quality of Cucumber Fruit

Agricultural waste composite substrates contain a variety of organic and inorganic nutrients, which can enhance crop quality. Soluble sugars, for instance, contribute to improved flavor by increasing the sweetness of fruits. Substrates used for tomato cultivation, compared to soil-based methods, have been shown to increase dry matter, sugar, and soluble solid content [44]. The application of earthworm manure, in particular, boosts chlorophyll content and carbohydrate and protein levels, thus improving fruit and seed quality [45].

In this study, as the proportion of mushroom residue increased and that of cattle manure decreased, the vitamin C and sugar content of cucumber fruit gradually increased, while the nitrate content decreased. These results suggest that an appropriate increase in mushroom residue can enhance fruit quality, which aligns with previous findings. The variations in fruit quality observed across the same treatment in different years may be attributed to changes in the cultivation environment.

Fatty acids serve as precursors for aromatic volatile compounds, and a higher linolenic acid content is favorable for enhancing cucumber aroma. The quality of cucumber fruit is determined not only by nutritional factors, but also by flavor, an important indicator of taste that is influenced by both the cultivation environment and genetic factors [33,46]. In this study, the use of different composite substrates significantly affected the content of aromatic compounds in cucumber fruit at the commercial stage. The increased mushroom residue content of the substrate led to higher levels of characteristic aromatic substances in the fruit, consistently with prior research. Therefore, improving cultivation substrates can be an effective strategy for enhancing the characteristic aroma of cucumbers. The ratios of mushroom residue and cattle manure notably influenced the total aroma content and the types and concentrations of various aromatic compounds in cucumber fruit. Further research is necessary to elucidate the mechanisms through which different substrate ratios affect volatile compound production.

## 5. Conclusions

This study explored the effects of different proportions of two agricultural waste-based substrates, i.e., cattle manure and mushroom residue, on the yield and quality of cucumber fruit. The different proportions of these mixed growing substrates significantly affected the physicochemical properties of the individual substrates and the yield and quality of cucumber fruit. The results showed that the T4 treatment (mushroom residue/cattle manure = 1:1) increased the yield and content of total sugar, Vc, and fatty acids of cucumber fruit, while the C6 aldehydes of cucumber fruit exhibited the highest aroma with the T6 treatment (mushroom residue/cattle manure = 3:1). An optimal mixed growing substrate could create a suitable growing environment for cucumber, effectively promoting its yield and quality.

## Figures and Tables

**Table 1:** Comparison of physicochemical properties of composite substrates with different proportions of cattle manure and mushroom residue. Different lowercase letters in same column represent significant differences at 0.05 level.

Treatment	Bulk Density (g/cm^3^)	Total Porosity (%)	Aeration Porosity (%)	Water-Holding Porosity (%)	Gas–Water Ratio (%)	pH	EC (mS/cm)
**CK**	0.22 ± 0.01 g	64.53 ± 0.89 a	17.43 ± 0.93 a	47.10 ± 0.31 ab	0.37 ± 0.02 a	5.93 ± 0.02 h	2.95 ± 0.02 c
**T1**	0.47 ± 0.00 a	52.03 ± 0.58 d	5.82 ± 0.10 f	45.37 ± 0.25 b	0.13 ± 0.01 e	6.90 ± 0.01 g	7.13 ± 0.02 a
**T2**	0.46 ± 0.00 a	52.22 ± 0.45 d	6.34 ± 0.18 e	45.88 ± 0.28 b	0.14 ± 0.00 e	6.96 ± 0.01 g	6.97 ± 0.02 b
**T3**	0.45 ± 0.00 b	53.28 ± 0.48 cd	6.68 ± 0.11 de	46.60 ± 0.37 b	0.14 ± 0.00 de	7.03 ± 0.02 f	6.65 ± 0.02 b
**T4**	0.42 ± 0.00 cd	53.86 ± 0.12 cd	6.59 ± 0.23 de	47.27 ± 0.12 ab	0.14 ± 0.01 e	7.09 ± 0.01 de	6.34 ± 0.02 a
**T5**	0.40 ± 0.00 de	54.80 ± 0.59 cd	7.32 ± 0.20 cde	47.48 ± 0.39 ab	0.15 ± 0.00 cde	7.14 ± 0.01 bc	5.31 ± 0.02 a
**T6**	0.39 ± 0.00 de	55.11 ± 0.74 c	7.46 ± 0.25 cd	47.66 ± 0.50 ab	0.16 ± 0.00 cde	7.18 ± 0.01 ab	4.73 ± 0.02 a
**T7**	0.39 ± 0.00 de	55.48 ± 0.31 b	7.65 ± 0.13 b	47.85 ± 0.30 a	0.16 ± 0.00 b	7.22 ± 0.02 a	3.33 ± 0.02 c

**Table 2:** Effects of different proportions of cattle manure and mushroom residue on cucumber yield. Different lowercase letters in same column represent significant differences at 0.05 level.

Treatment	Strain (kg)		Acre Yield (kg)	
	2020	2021	2020	2021
**CK**	0.76 ± 0.11 bc	2.53 ± 0.20 b	2123.83 ± 122.39 bc	3537.12 ± 280.93 b
**T1**	0.71 ± 0.07 c	2.83 ± 0.10 ab	1974.57 ± 184.47 c	3966.24 ± 136.37 ab
**T2**	1.05 ± 0.08 ab	2.63 ± 0.21 ab	2983.53 ± 221.25 ab	3684.83 ± 288.28 ab
**T3**	1.29 ± 0.08 a	2.53 ± 0.17 b	3603.98 ± 216.82 a	3535.57 ± 235.75 b
**T4**	1.04 ± 0.10 ab	2.95 ± 0.29 a	2913.66 ± 285.42 ab	4127.94 ± 404.92 a
**T5**	0.98 ± 0.14 bc	2.62 ± 0.16 ab	2733.30 ± 387.90 bc	3672.39 ± 380.54 ab
**T6**	0.80 ± 0.14 bc	3.06 ± 0.25 a	2226.44 ± 364.42 bc	4281.86 ± 224.59 a
**T7**	0.69 ± 0.07 c	2.99 ± 0.08 a	1918.60 ± 203.53 c	4179.24 ± 353.68 a

**Table 3:** Effects of different proportions of cattle manure and mushroom residue on sugar content of cucumber fruit. Different lowercase letters in same column represent significant differences at 0.05 level.

Years	Treatment	Fructose (mg/g)	Glucose (mg/g)	Sucrose (mg/g)	Total Content (mg/g)
**2020**	CK	7.06 ± 0.43 ab	9.50 ± 1.18 a	0.04 ± 0.00 ab	17.33 ± 1.73 a
	T1	6.02 ± 0.65 b	10.20 ± 0.46 a	0.03 ± 0.00 b	16.24 ± 1.07 ab
	T2	6.75 ± 0.25 ab	9.24 ± 0.91 a	0.05 ± 0.00 a	15.50 ± 1.22 ab
	T3	6.38 ± 0.41 ab	8.44 ± 1.03 a	0.05 ± 0.00 a	14.86 ± 1.37 ab
	T4	7.36 ± 0.23 a	10.23 ± 1.00 a	0.05 ± 0.01 a	17.63 ± 1.21 a
	T5	6.72 ± 0.21 ab	8.96 ± 0.88 a	0.05 ± 0.01 a	15.72 ± 1.04 ab
	T6	6.98 ± 0.24 ab	9.79 ± 0.98 a	0.05 ± 0.01 a	16.82 ± 1.22 ab
	T7	6.27 ± 0.35 ab	8.07 ± 0.86 a	0.06 ± 0.00 a	13.19 ± 1.13 b
**2021**	CK	8.65 ± 0.26 c	7.17 ± 0.14 d	0.06 ± 0.00 d	15.88 ± 0.40 c
	T1	9.02 ± 0.05 abc	7.40 ± 0.12 d	0.08 ± 0.01 abc	16.50 ± 0.170 c
	T2	9.35 ± 0.10 ab	7.27 ± 0.27 d	0.09 ± 0.01 ab	16.72 ± 0.31 bc
	T3	9.14 ± 0.20 abc	7.17 ± 0.11 d	0.07 ± 0.00 cd	16.38 ± 0.31 c
	T4	8.47 ± 0.08 c	8.65 ± 0.19 c	0.09 ± 0.01 ab	17.29 ± 0.20 bc
	T5	8.88 ± 0.39 abc	8.97 ± 0.20 bc	0.07 ± 0.00 bcd	17.92 ± 0.58 b
	T6	8.69 ± 0.22 bc	9.39 ± 0.25 b	0.07 ± 0.00 bcd	18.01 ± 0.55 b
	T7	9.51 ± 0.13 a	10.12 ± 0.33 a	0.10 ± 0.01 a	19.64 ± 0.69 a

**Table 4:** Effects of different proportions of cattle manure and mushroom residue on fatty acid content of cucumber fruit. Different lowercase letters in same column represent significant differences at 0.05 level.

Years	Treatment	Palmitic Acid (μg/g)	Stearic Acid (μg/g)	Linoleic Acid (μg/g)	Linolenic Acid (μg/g)
**2020**	CK	54.47 ± 2.78 ab	29.37 ± 1.95 abc	104.16 ± 1.46 cde	139.64 ± 9.46 c
	T1	47.73 ± 0.17 b	19.87 ± 1.39 c	85.26 ± 1.59 de	118.60 ± 2.04 c
	T2	54.18 ± 2.32 ab	23.26 ± 1.53 bc	117.63 ± 9.23 bcd	169.13 ± 8.35 bc
	T3	72.45 ± 17.95 ab	51.17 ± 20.44 a	69.14 ± 2.33 e	106.79 ± 1.29 c
	T4	76.10 ± 6.13 a	48.88 ± 10.87 ab	180.07 ± 15.12 a	255.74 ± 16.31 a
	T5	64.27 ± 5.90 ab	38.98 ± 1.95 abc	147.04 ± 19.49 abc	224.18 ± 29.79 ab
	T6	73.29 ± 4.02 a	45.67 ± 1.85 abc	177.37 ± 13.45 a	275.58 ± 26.54 a
	T7	64.24 ± 2.03 ab	42.67 ± 1.41 abc	158.01 ± 4.89 ab	260.94 ± 5.17 a
**2021**	CK	181.89 ± 5.23 d	60.60 ± 5.62 b	185.21 ± 5.76 d	407.39 ± 8.30 c
	T1	190.43 ± 2.96 cd	50.60 ± 1.70 b	231.75 ± 13.19 b	527.14 ± 26.09 b
	T2	190.24 ± 4.16 cd	50.25 ± 1.87 b	232.19 ± 5.90 b	544.02 ± 13.98 ab
	T3	211.86 ± 9.12 b	55.69 ± 3.40 b	259.48 ± 9.38 a	600.92 ± 25.72 a
	T4	192.42 ± 9.24 cd	56.54 ± 8.04 b	212.75 ± 5.78 bc	511.87 ± 19.59 b
	T5	207.14 ± 3.83 bc	54.10 ± 2.74 b	234.89 ± 5.63 b	588.98 ± 9.09 a
	T6	235.42 ± 2.96 a	84.26 ± 2.85 a	203.94 ± 6.73 cd	487.88 ± 15.57 b
	T7	189.57 ± 0.26 cd	53.94 ± 4.15 b	212.56 ± 9.70 bc	512.50 ± 24.67 b

## Data Availability

The original data of this study are included in the article. Data will be made available on request.
